# The Main Chemical Composition and *in vitro* Antifungal Activity of the Essential Oils of *Ocimum basilicum* Linn. var. *pilosum (Willd.)* Benth

**DOI:** 10.3390/molecules14010273

**Published:** 2009-01-08

**Authors:** Ji-Wen Zhang, Sheng-Kun Li, Wen-Jun Wu

**Affiliations:** 1 Institute of Pesticides, Northwest A&F University, Yangling, Shaanxi 712100, P.R. China; 2 College of Sciences, Northwest A&F University, Yangling, Shaanxi 712100, P.R. China; E-mails: zjwsmail1086@tom.com (J-W. Z.), saintkun001@163.com (S-K. L.)

**Keywords:** *Ocimum basilicum* Linn. var. *pilosum* (*Willd*.) Benth., Essential oil, Antifungal activity, Chemical composition

## Abstract

The essential oils of the aerial parts of *Ocimum basilicum* Linn. var. *pilosum* (*Willd*.) Benth., an endemic medicinal plant growing in China, was obtained by hydrodistillation and analysed by GC-MS. Fifteen compounds, representing 74.19% of the total oil were identified. The main components were as follows: linalool (29.68%), (*Z*)-cinnamic acid methyl ester (21.49%), cyclohexene (4.41%), α- cadinol (3.99%), 2,4-diisopropenyl-1-methyl-1-vinylcyclohexane (2.27%), 3,5-pyridine-dicarboxylic acid, 2,6-dimethyl-diethyl ester (2.01%), β-cubebene (1.97%), guaia-1(10),11-diene (1.58%), cadinene (1.41%) (*E*)-cinnamic acid methyl ester (1.36%) and β-guaiene (1.30%). The essential oils showed significant antifungal activity against some plant pathogenic fungi.

## Introduction

It is estimated that almost 30% of planted crops are lost due to attack of feeding insects, spoilage or disease [1]. Though chemical protection of plant plays an important role in ensuring sufficient food supply to a growing world population, in this era of increased concern about the safety of chemicals used in plant protection, natural methods of plant protection and natural preservatives are receiving increased attention, so nowadays, more and more attention is being paid to the use of natural products and their derivatives in terms of environment-friendly materials, negligible persistent residues good selectivity, and so on.

The harvest losses due to fungal disease in world crop protection may amount to 12% or even higher in developing countries [2]. Many pathogens including *Botrytis cinerea* (grey mold rot), *Fusarium oxysporum* (vascular wilt), *Sclerotinia sclerotiorum* (water soaked spot), *Fusarium solani* (fruit rot) and *Phytophthora capsici* (fruit rot) cause severe pre- and post-harvest damage to agriculture. Research focused on plant-derived fungicides and their possible application in agriculture is being intensified as these are having enormous potential to inspire and influence modern agro-chemical research. There is a good reason to suppose that the secondary metabolites of plants have evolved to protect them from attack by microbial pathogens [3]. For many years, a variety of different chemical and synthetic compounds have been used as antimicrobial agents to inhibit the plant pathogenic fungi. Antimicrobial chemicals such as benzimidazoles, aromatic hydrocarbons and sterol biosynthesis inhibitors are often used in control of plant disease in agriculture. However, there is a series of problems against the effective use of these chemicals in areas where the fungi have developed resistance. In order to overcome this problem, higher concentrations of these chemicals were used, but this increases the risk of high-level toxic residues in the products. Thus, there has been a growing interest on the research of the possible use of plant extracts, which can be relatively less damaging for pest and disease control in agriculture. In the past few years, due to concerns regarding the safety of synthetic antimicrobial agents there has been an increase in naturally developed substances, which has resulted in a huge increase in the use of naturally derived compounds as potential antifungal agents [4]. Within the diverse existing phytochemical groups, essential oils have widely proved their important antimicrobial properties [5]. 

*Ocimum (O.) basilicum* is an important medicinal plant and a culinary herb widely cultivated in many countries that contains several antioxidants compounds [6] and displays a high antioxidant power [7]. *O. basilicum* extracts have been shown to display important effects at cellular level, including the platelet anti-aggregant property and inhibitory activity against HIV-1 reverse transcriptase [8]. In addition, infusions of *O. basilicum* are used in traditional medicine to decrease plasma lipid content in some Mediterranean areas such as the Eastern Morocco. 

The herb *Ocimum basilicum* Linn.var. *pilosum (Willd.)* Benth., a popular garden and ornamental plant, is a member of the family *Labiatae*. The plant grows in the subtropical regions and is widely distributed throughout China [3]. All the aerial parts were used as a single effective prescription in folk medicine for treatment of cold, sedative. It was also used to clear heartburn, soothe the nerves, remove heat and eliminate toxins, as well as used in making perfume [9,10]. To the best of our knowledge, there are no published reports on the phytochemical composition and antifungal activity of the essential oil of this plant [11,12,13].

During our search for substances of antifungal activity from the secondary metabolites produced by plants, we have undertaken an investigation of chemical composition and antifungal activity into the essential oil of this species. Here, in this paper, we report the chemical composition and antifungal activity of the essential oils of this plant against *Fulvia fulva* (Cooke) Ciferri, *Glomerella cingulata* (Stonem.) Spauld. etSchrenk, *Alternaria alternate* (Fries : Fries) von Keissler*, Fusarium solani* var. *coeruleum* that are common pathogens in all the world [14]. 

The results of analysis of the yellow-green oils obtained by hydrodistillation are shown in Table 1; GC/MS analysis of the oils was done. Fifteen components representing 74.19% of *Ocimum basilicum* Linn. var. *pilosum (Willd.)* Benth. essential oil were characterized.

## Results and Discussion

### Chemical composition of the essential oils

As can be seen in Table 1, ten components were terpenes, three components belonged to the aromatic compounds class and two components among the fifteen components that were identified are from other groups. Two components in this herb, linalool and (*Z*)-cinnamic acid methyl accounted for the highest percentage. Some other components like cyclohexene, α-cadinol, diisopropenyl-1-methyl-1-vinylcyclohexane and 2,4--guaia-1(10),11-diene constitute a second tier of components of the essential oils.

**Table 1:** Chemical components of the essential oils of Ocimum basilicum Linn. var. pilosum (*Willd.*) Benth.

Retention time (min)	Molecular Formula	Compounds	Percentage (%)
2.60	C_6_H_10_	Cyclohexene	4.41
5.33	C_6_H_12_O_2_	2-Pentanone,4-hydroxy-4-methyl	0.69
13.85	C_10_H_18_O	Linalool	29.68
16.73	C_10_H_18_O	Terpinen-4-ol	0.49
21.00	C_10_H_10_O_2_	(*E*)-Cinnamic acid methyl ester	1.36
23.64	C_10_H_10_O_2_	(*Z*)-Cinnamic acid methyl ester	21.49
23.81	C_15_H_24_	Cyclohexane, 2,4-diisopropenyl-1-methyl-1-vinyl	2.27
25.27	C_15_H_24_	β-Guaiene	1.30
25.90	C_15_H_24_	1,4,7,-Cycloundecatriene,1,5,9,9-tetramethyl	0.56
26.69	C_15_H_24_	β-Cubebene	1.97
27.33	C_15_H_24_	Guaia-1(10),11-diene	1.58
27.51	C_15_H_24_	Caryophyllene	0.98
27.69	C_15_H_24_	Cadinene	1.41
31.41	C_15_H_26_O	α-Cadinol	3.99
34.65	C_13_H_17_NO_4_	3,5-Pyridinedicarboxylic acid, 2,6-dimethyldiethyl ester	2.01

### Antimicrobial activity of the essential oils

Results of the antimicrobial screening tests are shown in Table 2. As can be clearly seen in this table, we can easily conclude that the essential oils of *Ocimum basilicum* Linn. var. *pilosum (Willd.)* Benth. exhibited inhibitory activity against all the four tested fungi, especially towards *Fulvia fulva* (Cooke) Ciferri and *Fusarium solani* var. coeruleum, in which the EC_50_ is below 20 ppm.

**Table 2:** The effect of *O. basilicum* oil on *in vitro* growth of four phytopathogenic fungi.

Fungus	Toxicity regression equation	Correlation Coefficient	EC_50_ ppm
*Fulvia fulva* (Cooke)Ciferri	y=3.6900+1.0985x	r=0.8051	15.58
*Glomerella cingulata*(Stonem.)Spauld. etSchrenk	y=-3.4123+3.2843x	r=0.9595	363.92
*Alternaria alternate* (Fries : Fries) von Keissler	y=-3.8455+3.4343x	r=0.9561	376.70
*Fusarium solani* var. coeruleum	y=3.8228+1.0196x	r=0.7590	14.27

The results were obtained through the statistical software; Y is probit value transformed from inhibition rate. X is log(c) and c is the concentration of the sample.

## Experimental

### Collection of plant material

The aerial parts of *Ocimum basilicum* Linn. var. *pilosum (Willd*.) Benth. were collected from Tengzhou County, Shandong Province (East China) in September 2005 and identified by Mr Wu Zhenhai of Northwest A&F University [9]. The voucher specimen was deposited at the College of Sciences, Northwest A&F University. The aerial parts (leaves and flowers/inflorescences) were dried in the shade (at room temperature).

### Isolation of essential oils

The air-dried aerial parts of the plant (100 g) were powdered and the volatile fraction was isolated by hydrodistillation to give a yellow-green essential oil (0.43 g). 

### Gas chromatography-mass spectrometry analysis

The essential oil was analyzed by GC and GC/MS. GC-MS was performed with a Finnigan Trace DSQ GC-MS spectrometer (Thermo Company, US) employing the electron impact (EI) mode (ionizing potential 70 eV) and a capillary column (30 m × 0.25 mm, film thickness 0.25 μm) packed with 5% phenyldimethylsilicone on HP-5 (Hewlett-Packard, Palo Alto, CA). Ion source temperature was 280 °C. The GC settings were as follows: the initial column temperature was set at 40 °C and held isothermal for 1 min; the temperature was programmed from 40 to 220 °C at a rate of 3 °C/min, and was kept 220 °C for 25 min, then improved to 280 °C at 5 °C/min and hold for 10 min. The oven temperature was 280 °C. Helium was used as the carrier gas, flow rate 1 mL/min. Split ratio, 1:50.

### Identification of components

The percentage composition of the samples was computed from the GC peak areas. Library searches were carried out using the Wiley GC/MS Library.

### Assessment of antimicrobial activity

The tested pathogenic fungi: *Fulvia fulva* (Cooke)Ciferri, *Glomerella cingulata*(Stonem.)Spauld. etSchrenk, *Alternaria alternate* (Fries : Fries) von Keissler*, Fusarium solani* var. *coeruleum* were provided by the Institute of Pesticides, Northwest A&F University. The essential oil dissolved in acetone was screened for antifungal activity *in vitro* by measurement of inhibitory zone diameter, a Poisoned Food Technique. The general procedure goes as follows:

Cultures of the test fungus were maintained on potato-dextrose-agar (PDA) medium slants at 25°C, and were subcultured in Petri dishes prior to testing. The ready-made medium (39 g) was suspended in distilled water (1000 mL) and heated to boiling until it had dissolved completely. The medium and the Petri dishes were autoclaved for 30 min. Stock solutions were prepared by dissolving the test materials in acetone, and diluted to five different concentrations. The five different final concentrations of the oil was 1000, 500, 250, 125, 62.5 ppm respectively. Acetone was the solvent to help the oil diluted into the PDA medium. Acetone was served as control. The medium was poured into a set of two Petri dishes (two replicates) under aseptic conditions in a laminar flow chamber. When the medium in the plates was partially solidfied, a 5-mm thick disc of fungus (spores and mycelium) cut from earlier subcultured Petri dishes was placed at the centre of the semi-solid medium and the lids of the dishes were replaced. The treated and control dishes were kept in a incubator at 26 (±2) °C till the fungal growth in the control dishes was almost complete (2±3 days) [15].The mycelial growth of fungus (mm) in both treated (T) and control (C) Petri dishes were measured diametrically in three different directions and the growth inhibition (I) was calculated using the formula:

I(%)= {(C-d)-(T-d)}/(C-d) ×100

Where d: Diameter of the cut fungus, C: Diameter of the control fungus, T: Diameter of the treated fungus (measurement unit:mm, two colonies were counted in two dishes. It was repeated for three times)
